# Non-Melanoma Skin Cancer: Assessing the Systemic Burden of the Disease

**DOI:** 10.3390/cancers17040703

**Published:** 2025-02-19

**Authors:** Emmanouil Karampinis, Dimitra Koumaki, Dimitrios Sgouros, Paraskevi-Maria Nechalioti, Olga Toli, Georgia Pappa, Marios Papadakis, Konstantina-Eirini Georgopoulou, Angeliki-Victoria Schulze-Roussaki, Demetrios Kouretas

**Affiliations:** 1 Second Dermatology Department, School of Health Sciences, Aristotle University of Thessaloniki, 54124 Thessaloniki, Greece; 2 Department of Dermatology, Faculty of Medicine, School of Health Sciences, University General Hospital of Larissa, University of Thessaly, 41110 Larissa, Greece; roussaki@otenet.gr; 3 Department of Dermatology, University Hospital of Heraklion, 71500 Crete, Greece; dkoumaki@yahoo.gr; 4 2nd Department of Dermatology and Venereology, “Attikon” General University Hospital, Medical School, National and Kapodistrian University of Athens, 12462 Athens, Greece; dimitris.sgouros.dermatology@gmail.com (D.S.); gpappa100@gmail.com (G.P.); 5 Department of Biochemistry and Biotechnology, University of Thessaly, Viopolis, Mezourlo, 41500 Larissa, Greece; marianechalioti98@gmail.com; 6 Department of Dermatology, Oncoderm Center One Day Clinic, 45332 Ioannina, Greece; olgatolimail@gmail.com; 7 Department of Surgery II, Witten/Herdecke University, Heusnerstrasse 40, 42283 Witten, Germany; marios_papadakis@yahoo.gr; 8 Department of Dermatology, General Hospital of West Attica “Agia Varvara”, 12351 Athens, Greece; koneirgeo@gmail.com

**Keywords:** non-melanoma skin cancer, BCC, SCC, oxidative stress, redox status, inflammation, interleukins, immunosuppression, systemic disease

## Abstract

The emergence of systemic therapies and photoprotection against non-melanoma skin cancer (NMSC) raises questions on the broader systematic impact of the disease. Personalized medicine involves a holistic patient approach, through which the evaluation of systemic biomarkers can reveal the interconnected aspects of patient health and tailored therapies. Cumulative UV exposure disrupts redox equilibrium and triggers inflammation and cutaneous immunosuppression, processes that contribute independently or via their interplay to cutaneous carcinogenesis. This systemic impact can be further reinforced by biomolecules derived from the NMSC microenvironment, fueling a continuous cycle of oxidative stress and inflammation in the organism. Regarding investigation of the systemic burden of NMSC, we conducted a narrative review focusing on parameters related to redox status, inflammation, and immune suppression observed in the blood components (serum, plasma, and erythrocytes) of NMSC patients. Our findings revealed an association of NMSC patients with perturbations of redox homeostasis, as evidenced by the decreased antioxidant activity, lower levels of non-enzymatic antioxidants, and increased byproducts of lipid, protein, and DNA oxidative damage. Additionally, NMSC patients presented augmented levels of pro-inflammatory interleukins, reduced anti-tumor biomolecule levels, and enhanced immune response markers, as well as elevated vitamin D levels. These systemic changes may lead to the association of NMSC with a higher risk of secondary malignancies in other organs. Overall, the findings of the present study suggest that NMSC affects systemic health beyond the skin, underscoring the need for a comprehensive and individualized approach to the management and monitoring of the patient.

## Simple Summary

Non-melanoma skin cancer (NMSC), strongly associated with prolonged ultraviolet (UV) radiation exposure, is the most prevalent type of skin cancer in Caucasians. The present review article summarizes the available evidence on the impact of NMSC incidence and cumulative sun exposure on systemic homeostasis, particularly focusing on oxidative stress, inflammation, and immune system response. Understanding these systemic alterations is crucial for unraveling the systemic burden of NMSC beyond skin damage. This complex interplay highlights the need for comprehensive care, including systemic therapies, effective photoprotection, and the potential role of targeted nutritional interventions to support overall health.

## 1. Introduction

Skin cancer encompasses melanoma and non-melanoma skin cancer (NMSC), the latter of which comprises basal cell carcinoma (BCC), cutaneous squamous-cell carcinoma (SCC), as well as several less common skin tumors. BCC develops from the basal layer of the epidermis and its appendages, while SCC arises from the malignant proliferation of atypical epidermal keratinocytes [1,2]. Although skin cancer is among the most common cancers globally, research on its various types and prevalence across different continents, particularly those with fewer white-skinned populations, remains limited. Australia and New Zealand have the highest incidence rates of skin cancer, while Europe records the highest incidence and mortality rates for melanoma skin cancer. In contrast, North America and Asia report the highest incidence and mortality rates for non-melanoma skin cancers [3,4]. Ultraviolet radiation (UV) is the primary environmental risk factor for skin cancer development. The rising risk of NMSC is linked to the aging population, fair skin, and increased occupational and recreational UV exposure, as well as certain lifestyle choices such as tanning. Immunosuppression, and particularly the immunosuppressive medications patients take to prevent organ rejection in the case of transplant patients, elevate the risk of more aggressive NMSC occurrence. Also, agents such as arsenic acid can act as a co-carcinogen by enhancing the cytotoxic and mutagenic effects of UV radiation on cells, increasing the risk of cancer development [4]. Early detection is the key factor in successfully treating skin cancer, with advancements in technology continually improving methods for identifying the disease at its earliest stages [5].

Ultraviolet radiation (UV) serves as the main environmental risk factor for NMSC, inducing several types of DNA damage and subsequent mutations. UV-induced damage can be ascribed to reactive oxygen species (ROS) production, the disturbance of redox homeostasis, and inflammation and cutaneous immunosuppression, processes that promote cutaneous carcinogenesis individually or interconnected with self-perpetuating and vicious circles [6,7,8].

This series of events seems to have a greater impact beyond the skin, as UV-induced damaged biomolecules can enter the bloodstream and lead to systemic oxidative stress and inflammation. Additionally, UV radiation has been implicated in cutaneous immunosuppression, mediated by Langerhans cell depletion and a reduction in epidermal antigen-presenting cell function, processes that increase the skin’s vulnerability to carcinogenic stimuli [9]. These modifications are anticipated to influence the levels of several biomarkers in biological specimens (plasma, serum, saliva, and urine), creating a distinct systemic profile of NMSC patients. Notably, oxidative stress can alter this systemic profile by depleting antioxidant defenses. This mechanism reflects an attempt of the human body to counteract the excessive ROS levels and the subsequent oxidative damage [10].

Moreover, the microenvironment of NMSC can be an additional site of ROS and the production of inflammatory mediators. In BCC and SCC cases, cancer cells display an aberrant redox state due to the rapid energy metabolism and accelerated proliferation that characterize tumor cells, resulting in ROS production. The induced oxidative stress damages the surrounding tissues and facilitates cancer expansion. In addition, cancer cells themselves favor tumor progression, invasion, and metastasis, producing certain inflammatory biomolecules that can travel to neighboring tissues and enter the systemic circulation via microcirculation, thereby having a broader impact not just limited to the skin [10].

Within this context, NMSC patients are expected to present distinct differences in certain systemic parameters compared to healthy individuals with limited UV exposure. Therefore, our study aims to review the existing data on these parameters in NMSC patients and healthy people, with the overall object of elucidating the effects of cutaneous carcinogenesis on patients’ overall health.

### 1.1. Systemic Oxidative Stress

Oxidative stress refers to the imbalance between ROS production and the biological system’s capacity to detoxify these highly reactive molecules. The term ROS encompasses both radical and non-radical oxygen derivatives, formed through the partial reduction of oxygen, including superoxide anions (O^2•−^), hydrogen peroxide (H_2_O_2_), and hydroxyl radicals (HO•). Assessment of oxidative stress is performed either through the direct measurement of ROS or the determination of oxidative damage and antioxidant status, enzymatic and non-enzymatic. Typically, the oxidative stress response of test subjects is compared to a control group that has not been exposed to the oxidative factor [11].

**Figure 1:**
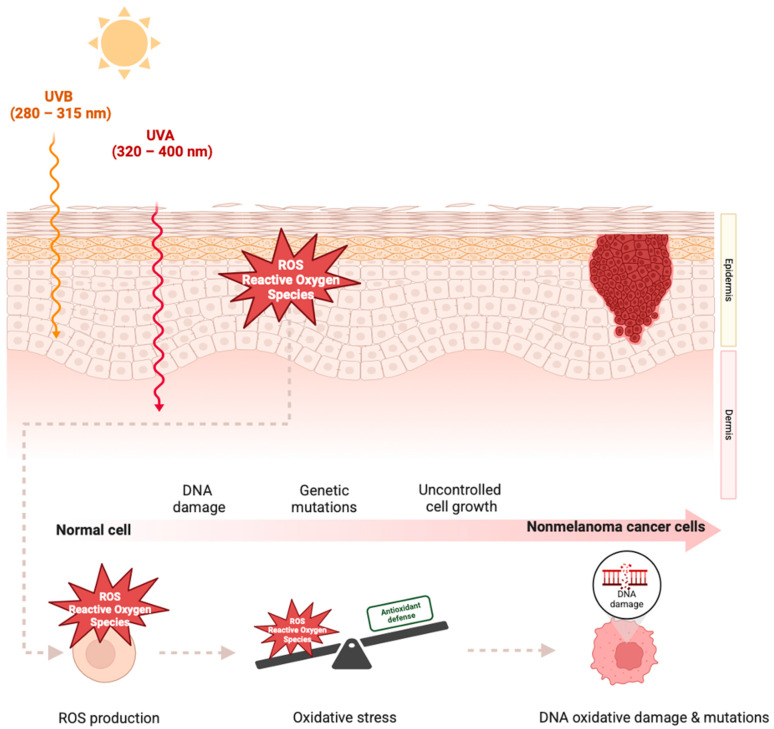
Cutaneous oxidative stress disturbance can promote skin carcinogenesis. The UV-induced ROS lead to cutaneous antioxidant enzyme consumption, weakening the cutaneous “antioxidant wall”. The prevalence of ROS finally leads to DNA oxidative damage and mutations leading to skin cancer formation. This figure has been created with BioRender.com.

The UVA range (320–400 nm), with some overlap in the UVB range (280–315 nm), is the primary external source of ROS and can contribute to cutaneous oxidative stress. This phenomenon occurs when photons are absorbed by intrinsic photosensitizers such as cytochromes, riboflavin, heme, and porphyrin, resulting in ROS generation and oxidative damage (Figure 1) [12]. Oxidative stress can be localized in a specific area, indicating a disease-specific context, like the microenvironment of cancerous tissues or psoriasis plaques. Interestingly, oxidative stress can affect the surrounding tissues, establishing a broader impact. In BCC patients, localized oxidative stress is assessed within the cancer tissue and the surrounding skin, illustrating the contribution of oxidative stress to cancer progression, particularly in areas exposed to the same stressor, such as UV radiation. Typically, oxidative stress parameters in the cancerous tissue are elevated compared to those in the adjacent areas. Also, the surrounding skin exhibits a more disrupted redox balance than normal skin, likely due to the direct impact of oxidative stimuli and the transfer of oxidants from the nearby tumor [13].

On the contrary, systemic oxidative stress involves the assessment of redox parameters in various biological specimens such as serum and plasma, urine, or saliva, providing insight into the overall redox status of the organism. The concept of systemic oxidative stress is closely linked to the exposome, including all environmental exposures and individual encounters throughout the patient’s life, such as diet, physical activity, and lifestyle choices. These factors, along with genetic influence and internal characteristics like the microbiome and metabolism, significantly affect an individual’s oxidative stress response [9].

Differences in oxidative stress parameters can be detected when comparing localized and systemic oxidative stress, probably attributed to the intensity, type, and target of the stressor, between local tissues and the systemic environment. More specifically, in localized oxidative stress, specific tissues may present high ROS levels as a direct result of UV exposure. In contrast, systemic oxidative stress encompasses the cumulative effect of various stressors circulating throughout the body, and parameters such as aging, comorbidities, and systemic medications [9]. Moreover, the impact of a stressor can be influenced by the “default antioxidant wall” of the disturbed system. As the stressor is distributed through the body, it is countered by the intact antioxidant mechanisms of other systems, preventing systemic oxidative stress. However, this preconditions the existence of a well-functioning antioxidant system. Consequently, the effect of a localized stressor such as UV in patients with already diminished systemic antioxidant defenses (aging, malnutrition, etc.) is greater. Therefore, examining systemic oxidative stress markers in patients with skin cancer is crucial for guiding patient treatment.

### 1.2. Systemic Inflammation

Apart from systemic oxidative stress, the systemic profile of NMSC patients is also affected by systemic inflammation. Similarly to oxidative stress, the onset of systemic inflammation often originates from the skin, where the initial damage occurs. One notable effect of UV exposure is the upregulation of cyclooxygenase-2 (COX-2) in keratinocytes, leading to the increased production of prostaglandin E2 (PGE2), which in turn causes inflammation in the skin tissue. Cutaneous inflammation, mainly in the form of a chronic subtype, can lead to DNA damage and genomic instability, which may include mutations in critical genes responsible for cell cycle regulation and apoptosis [14].

**Figure 2:**
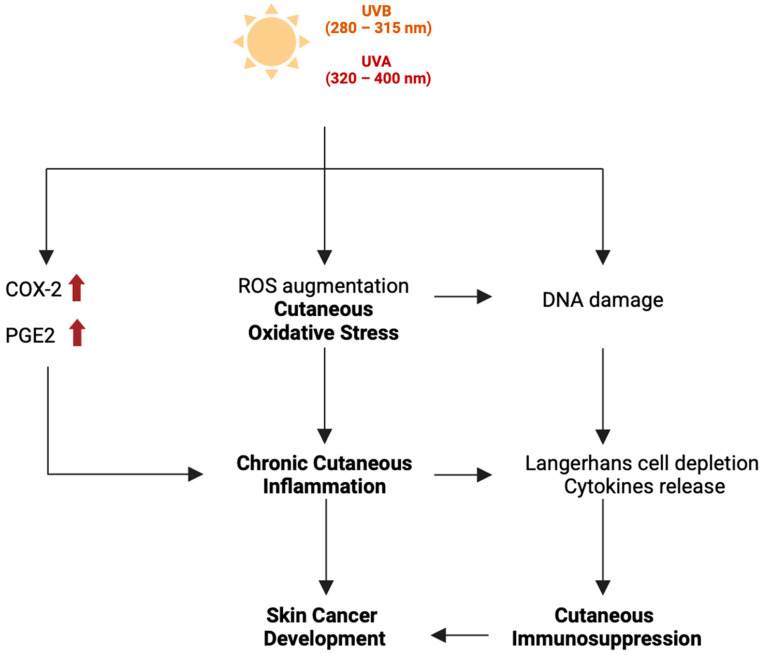
Interplay between UV-induced oxidative stress, chronic inflammation, and immunosuppression in the skin. This figure has been created with BioRender.com.

Following keratinocyte activation, these cells secrete immune-related molecules, including growth factors, cytokines, and chemokines, which modify the local microenvironment and heighten susceptibility to inflammation and subsequent tumor development (Figure 2). In addition to keratinocytes, activated fibroblasts can produce and deposit collagen, causing fibrosis and establishing a pro-inflammatory microenvironment in NMSC. The excessive production of those inflammatory factors can trigger a systemic inflammatory response, causing a sharp increase in neutrophils. Simultaneously, some of these cytokines may induce immunosuppression, leading to the apoptosis of numerous lymphocytes. This process weakens the host’s ability to manage immune responses effectively, thereby increasing susceptibility to further dysregulation [15].

As indicated by the concept of oxidative stress, localized and systemic inflammation can differ significantly due to the direct inflammatory impact of the stimuli on the on-focus tissue. The systemic immune alterations, aside from direct induction by UV radiation and cancerous tissue, can be affected by oxidative mechanisms, an interaction creating a detrimental vicious circle between cellular redox status and inflammation response. This cyclical relationship is particularly crucial in NMSC patients, where both oxidative stress and inflammation contribute to disease progression. During inflammation, the functions of immune cells can be altered, turning into phenotypes characterized by an increased production of both inflammatory transmitters and ROS. This shift facilitates the perpetuation of chronic inflammation, which can further exacerbate oxidative damage to the genome [15,16]. Also, many inflammation inducers, particularly pro-inflammatory cytokines, are known to have indirect oxidative effects, such as, for example, TNF-α, which has been associated with the pro-inflammatory differentiation of macrophages, which can result in excessive ROS generation [17].

### 1.3. Biochemical Profile and Metabolism

Chronic UV-induced oxidative and inflammatory responses have profound effects on the biochemical and metabolic profiles of NMSC patients. The inflammatory markers released after UV exposure can interfere with insulin signaling pathways, causing insulin resistance and disturbances in glucose and lipid metabolism [18], as well as an increase in the markers of functional inadequacy in multiple organs, indicating the systemic impact of the condition [19]. Moreover, chronic systemic oxidative stress in patients with NMSC creates an environment in which antioxidants are rapidly depleted, resulting also in vitamin and nutrient insufficiency and deficiency. For example, studies indicate that NMSC patients frequently show folate deficiency, likely because folate—known for its antioxidant role—is extensively used to counteract heightened ROS production [20]. Also, there are unusual cases of chronic iron deficiency anemia in individuals with neglected BCCs associated with micro bleeding and chronic inflammation. The mechanical deficits of the skin brought about by the cancer can affect the systemic biochemic profile and alter the complete blood count [21]. Poor nutrition, which is usually characteristic of patients with NMSC, further exacerbates these biochemical imbalances, with many cases exhibiting significant micronutrient deficiencies [22].

Therefore, comparing the biomarkers of redox status, systemic inflammation, and the biochemical and metabolic profiles of individuals with non-melanoma skin cancer (NMSC) and healthy individuals can provide valuable insights into the systemic implications of the disease. These comparisons can help clarify whether NMSC operates solely as a localized skin condition, or if it has more extensive effects on overall physiological health. This approach can also reveal how a medical history of intensive UV exposure influences biological processes beyond the skin, paving the way for new preventive strategies and targeted interventions that address both the cutaneous and systemic risks associated with NMSC.

## 2. Methodology

To conduct our narrative review, we identified the key terms commonly used in the literature to evaluate the oxidative stress and systemic inflammation parameters and biochemical and metabolic profiles in NMSC patients. Assessment of oxidative stress was performed based on a comprehensive battery of biomarkers. This battery included enzymatic and non-enzymatic antioxidant molecules, such as metabolic antioxidants and micronutrients, as well as markers of oxidative damage (Table 1). The direct measurement of ROS cellular levels and the balance of oxidants and antioxidants is one approach to determining the oxidative conditions. Oxidative stress can also be assessed indirectly, by measuring the indicators of oxidative modifications to significant biomolecules such as DNA/RNA damage, lipid peroxidation, and protein oxidation [9,23].

As for inflammation and immunosuppression, we evaluated a panel of markers reflecting both acute and chronic immune responses, including acute inflammatory markers, pro-inflammatory cytokines, blood count parameters, soluble immune checkpoints, and biomolecules associated with immunosuppression, as well as markers of heightened immune activity, such as antimicrobial peptides [24]. Acute inflammatory markers and pro-inflammatory cytokines are expected to multiply after acute UV exposure, with questions arising on their behavior following chronic UV exposure. Cytokines, often recognized as disease-specific indicators, play a critical role in modulating inflammatory and immune responses. However, little is known about their action in NMSC patients [25].

We investigated the aforementioned parameters in NMSC patients, taking into consideration the type of sample used (plasma, serum, urine, or saliva), as well as the protocol performed in each assessment. Each specimen has unique biochemical properties that can influence the observed levels of biomarkers and provide distinct perspectives on the same parameter [26]. For instance, while plasma and serum are both derived from whole blood, they are processed differently, resulting in distinct biochemical profiles. Serum is the liquid fraction collected after blood is allowed to clot; during coagulation, the fibrin clots formed by blood cells and related coagulation factors, are separated from the serum through centrifugation. In this process, platelets release various proteins including pro-inflammatory cytokines and metabolites into the serum. On the contrary, plasma is collected by adding an anticoagulant such as EDTA or heparin to the whole blood to prevent clotting before the blood cells are separated, allowing them to remain intact. Consequently, plasma is often favored for assessing overall antioxidant status compared to serum, reflecting the antioxidant levels across all blood components. However, the precise differences in antioxidant profiles between plasma and serum remain unclear [26]. Interestingly, a recent study found that plasma samples showed higher resistance to oxidative stress, whereas serum displayed a greater ability to scavenge ABTS cation radical, likely due to the presence of proteins, such as albumin, that contribute significantly to its antioxidant properties [6]. In the case of metabolite concentration, serum typically presents higher levels, due to the deproteinization of the sample, which eliminates the volume occupied by proteins, creating a more concentrated environment for low-molecular-weight compounds. For this reason, the metabolites in serum occupy a reduced volume, leading to a higher concentration, which enhances the sensitivity of biomarker detection in serum samples [27]. Differences in cytokine concentrations in serum and plasma were also reported [28].

Interestingly, saliva has become a popular diagnostic fluid for biochemical assessments due to its convenient and non-invasive nature. However, this is not the case for inflammatory markers such as cytokines, since the oral cavity represents a distinct environment influenced by local immune processes [29]. Urinary biomarkers can also be affected by the local environment. To maintain consistency and relevance in our study, we excluded studies that evaluate urine or saliva as the sole source of systemic biomarkers for comparison. Additionally, we excluded markers highly influenced by dietary intake, such as macro- or/and micronutrients, which may not reflect directly the biochemical disruptions specific to UV exposure [9].

In this review, we summarized the systemic alterations detected in NMSC patients, in order to elucidate their potential implications in the systemic impact of NMSC. We searched PubMed articles published before the end of September 2024, based on the terms of Table 1, Table 2 and Table 3, adding terms [BCC] OR [SCC] OR [NMSC]. Studies were included if they assessed systemic biomarkers related to oxidative stress, inflammation, or immunosuppression in NMSC patients, and were peer-reviewed and published before September 2024. Only studies analyzing systemic samples derived from blood (e.g., serum, plasma, and erythrocytes) were considered. Studies were excluded if they focused on histopathological or tissue-based biomarkers, cutaneous biomarkers without systemic evaluation, dietary biomarkers or antioxidant supplementation effects, pediatric populations or animal models, or lacked sufficient methodological details regarding biomarker assessment.

## 3. Comparative Datasets of NMSC Patients and Healthy Individuals

Our review identified a total of 10 comparisons of systemic oxidative stress levels in BCC patients and healthy individuals. Among these studies, five reported enzymatic mechanisms, five included non-enzymatic antioxidants, and four referred to oxidative damage to DNA, lipids, and proteins. In the case of SCC patients, six studies were reported, of which one involved the enzymatic activity of catalase, while two referred to non-enzymatic antioxidant mechanisms, and the other three studies to oxidative stress byproducts.

Specifically, enzymes such as catalase, glutathione peroxidase (GPx), and superoxide dismutase (SOD) are reported as possible redox biomarkers of NMSC patients. Catalase is an enzyme typically found in peroxisomes, serving a critical role in breaking down H_2_O_2_, while SOD catalyzes the reaction that converts superoxide radicals (O_2_^•−^) into less reactive molecules. In contrast, glutathione peroxidases (GPx) are a group of enzymes that reduce various organic and inorganic hydroperoxides to their corresponding hydroxyl compounds, using glutathione as a reducing agent [30]. Decreased enzymatic activity signifies a reduction in antioxidant defense mechanisms, a finding commonly observed in many cases of NMSC incidence.

Regarding non-enzymatic antioxidant compounds, only one comparison was reported without reaching statistical significance (GSH in erythrocytes in SCC patients). It is worth mentioning the fact that the protocol used in this study was adjusted for the hemoglobin concentration, a varying parameter for cases of anemia [31]. Assessment of oxidative damage, including lipid peroxidation (TBARS, MDA), protein carbonylation (CARBS), and DNA damage, indicated elevated levels in all studies, suggesting significant redox perturbations in NMSC patients. In most cases (Table 4) and comparisons (only two without association found) reviewed, there was a clear indication of an impaired redox status (reduced enzymatic and non-enzymatic antioxidant defense and increased oxidative stress byproducts). Most studies propose that this imbalance is primarily caused by the indirect effects of UV exposure, rather than the direct escape of ROS from the NMSC microenvironment [12,13]. Consequently, systemic oxidative stress has emerged as a defining feature in patients with NMSC, reflecting the systemic impact of the disease.

In the case of systemic inflammation, we detected two studies about acute-phase inflammation markers (CRP, ceruloplasmin), five about serum interleukin concentration, one about soluble immune mediators, and one about antimicrobial peptides (Table 5). The studies focused on acute inflammation showed diverse results regarding ceruloplasmin’s role, highlighting its dual antioxidant and anti-inflammatory properties. Indeed, ceruloplasmin, a serum ferroxidase enzyme, serves a critical role in plasma. By binding copper and catalyzing the oxidation of Fe^2+^ (ferrous iron) into Fe^3+^ (ferric iron), ceruloplasmin reduces the availability of free iron, which can eventually generate harmful free radicals [45]. The fact that all markers discussed both in Table 2 and Table 3 are interconnected and can influence the vicious circle between oxidative stress disturbance and inflammation is worth mentioning.

Assessment of interleukin concentrations in NMSC patients revealed some intriguing findings, as evidenced by the increased and decreased levels of specific interleukins. Notably, IL-17, a strong pro-inflammatory cytokine [49], was found to be increased, while Il-2, an anti-tumor interleukin, had decreased in patients [47,48] (Table 5). Moreover, an increase in IL-10 levels, an immunosuppressive cytokine, was reported [48]. These alterations in interleukin levels might suggest a unique “IL status” in NMSC patients, with most studies attributing this observation to increased cytokine production by skin cancer cells and diminished anti-tumor immunity (Table 5). This phenomenon is particularly evident in BCC, which is characterized by a Th2-skewed immune response and an increase in cytokines that promote tumor growth [39]. Analysis of the systemic inflammatory response revealed increased levels of four different soluble inflammatory mediators and antimicrobial peptides, such as defensin- β and cathelicidin [53], reinforcing in this way the association of NMSC with systemic inflammatory changes (Table 5).

Studies have reported correlations between serum inflammatory markers and the size and number of NMSC lesions present in patients. For example, patients with tumors larger than 1 cm presented lower mean IL-2 levels compared to those with smaller ones. Also, correlations between CRP and soluble immunology mediators were detected, reporting their interlinked roles in inflammatory and immune responses to NMSC. Interestingly, serum markers such as defensin-β have been highlighted by researchers as potential diagnostic biomarkers in BCC.

Table 4 and Table 5 demonstrate the complex interplay between systemic oxidative stress and inflammation present in NMSC patients. When observing the high prevalence of BCC, the most common skin cancer in Caucasian populations, questions arise on the impact of such biochemical imbalances on the systemic blood profiles of these patients. However, studies focusing on lipid metabolism reported inconsistent findings (Table 6). When assessing liver and renal function, elevated markers were reported in NMSC patients compared to non-NMSC patients (except for γ-GT), but were within the normal limits. This trend was present in most cases across various organ-related markers, indicating no specific organ dysfunction [19]. The studies focusing on lipid profiles have been performed primarily on BCC patients (Table 6).

The relationship between vitamin D and NMSC patients has been an extensively controversial topic, with numerous systematic reviews and meta-analyses investigating this complex association. Although this specific review does not focus on this controversy, but on the systemic impact of UV exposure and NMSC occurrence, it is valuable to consider the findings of Seretis et al.’s work [55], a comprehensive umbrella review that concluded that NMSC had higher vitamin D than controls (Table 6). Studies have also shown that elevated vitamin D levels are associated with increased calcium concentrations in many NMSC patients, as vitamin D3 stimulates calcium absorption through the gut [56].

**Table 6:** Comparisons of systemic biochemical and metabolic parameters in NMSC patients compared with control subjects.

Study Focused on BCC Patients	Parameter	Results	Study Focused on SCC Patients	Parameter	Results
**Glucose metabolism**					
[19]	Fasting glucose	Higher	[22]	Fasting glucose	Higher
**Lipid metabolism**			**Liver function**		
[57]	LDL	No association	[19]	ALT	Higher
[58]	LDL	No association	[19]	AST	Higher
[57]	HDL	No association	[19]	γGT	Lower
[58]	HDL	Lower	[19]	Total bilirubin	Higher
[57]	TGs	No association	**Renal Function**		
[58]	TGs	No association	[19]	Urea	Higher
[59]	TGs	No association	[19]	Creatinine	Lower
[57]	Total cholesterol	No association	**Hormones**		
[58]	Total cholesterol	Higher	[55]	Vit-D-25	Higher (based on umbrella metanalysis)
[59]	**Total cholesterol**	**No association**	**Electrolytes**		
[59]	**Total lipids**	**Higher**	[60]	Calcium	Higher
[59]	**Phospholipids**	**Higher**	[61]	Calcium	Higher
**Hormones**			[62]	Calcium	Higher
[55]	Vit-D-25	Higher (based on umbrella metanalysis)			
**Liver function**					
[19]	ALT	Higher			
[19]	AST	Higher			
[19]	γGT	Lower			
[19]	Total bilirubin	Higher			
**Renal Function**					
[19]	Urea	Higher			
[19]	Creatine	Lower			

## 4. Total Overview of the Systemic Profile of NMSC Patients

**Figure 3:**
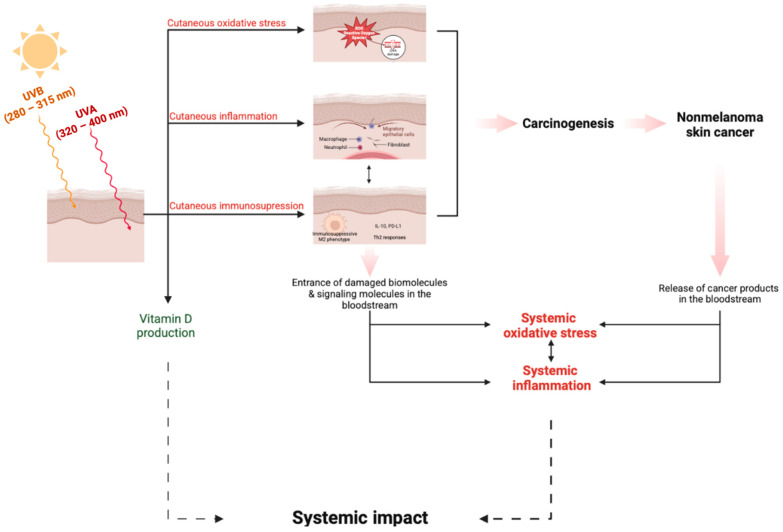
The dual contribution of chronic UV-induced damage and established NMSC to systemic oxidative stress and inflammation, creating a circular relationship between these processes: pathway A—entrance of biomolecules arising from the chronic UV-damaged skin and pathway B—entrance of biomolecules and mediators arising from the NMSC microenvironment. A noteworthy systemic effect associated with NMSC is the elevated levels of vitamin D often observed in patients, particularly compared to healthy controls, which can, in turn, increase the risk of hypercalcemia. This figure has been created with BioRender.com.

The above findings indicate that patients with NMSC indeed present implications that extend beyond the localized skin tumor, leading to systemic oxidative stress and systemic inflammation. As shown in Figure 3, exposure to intense UV radiation plays a critical role in initiating skin carcinogenesis (pathway A). This UV-induced pathway not only contributes directly to skin damage, but also sets off systemic consequences, as oxidative stressors and inflammatory agents enter the bloodstream. The microenvironment of the tumor (pathway B) further amplifies this effect by releasing additional agents that are both oxidative and inflammatory in nature, promoting a feedback loop of inflammation and oxidative stress. Interestingly, the one-direction perpetuating pathway B has also been confirmed through studies measuring oxidative stress and inflammation markers, particularly pre-inflammatory cytokines such as IL-17 and IL-23, before and after tumor excision or destruction [10,60,63]. While both studies proved these parameters with tumor removal, they did not reach healthy individuals’ systemic condition levels, probably due to the impact of pathway A. Studies focused on patients with actinic keratosis reinforce the hypothesis of pathway A, reporting induced oxidative stress in individuals with cumulative sun exposure, however, without reaching the stage of carcinogenesis [36]. These changes provide insights into other systemic observations among NMSC patients, such as elevated but within-normal-range organ function, as well as increased susceptibility to presenting other type of malignancies, with the inflammation–oxidative vicious circle being the etiology of many types of carcinogenesis (Figure 3) [64]. Also, it is notable that in the case of NMSC patients, higher systolic and diastolic blood pressure was displayed compared to controls, suggesting further systemic manifestations of those patients [19].

Increased vitamin D is another systemic characteristic of NMSC patients. Vitamin D plays a critical role in calcium metabolism, and as a result, patients with NMSC exhibit a lower risk of fractures and bond abnormalities. However, excessive levels of vitamin D and calcium can lead to symptoms of hypercalcemia and manifest as fatigue or gastrointestinal manifestations [56]. The hypothesis of hypercalcemia due to the excessive production of parathyroid hormone-related protein by tumors has been assessed in studies, with researchers concluding that it is indeed frequent in the case of SCC patients [60]. Also, as observed in the study of Karampinis et al., vitamin D deficiency can be reported in NMSC patients, especially in the case of elderly NMSC patients that avoid sun exposure. This deficiency is likely exacerbated by the induced systemic oxidative stress, which contributes to a greater deficiency compared to healthy individuals with low vitamin D levels [32].

Giving a total overview of the systemic profile of NMSC patients based on our findings, blood component analyses (including plasma and serum) reveal distinct patterns associated with the condition:

(a) Decreased antioxidant defense (lower enzymatic activity and non-enzymatic antioxidant levels);
(b) Increased oxidative damage byproducts of lipids, proteins, and DNA;
(c) Increased pro-inflammatory interleukins;
(d) Decreased anti-tumor biomolecule concentrations;
(e) Increased immunologic response compounds;
(f) Increased vitamin D levels;
(g) Hypercalcemia.

It is essential to emphasize that these findings were associated with UV-related NMSC carcinogenesis. While UV radiation is a predominant cause of NMSC, skin cancer can arise from other less common factors, such as pesticides and skin-toxic chemicals. A study focusing on arsenic-induced skin cancer reported distinct oxidative stress markers [65].

It is worth mentioning that in the context of genetic syndromes such as Gorlin syndrome and multiple keratoacanthoma syndrome, redox and inflammation markers are probably different, as the genetic abnormalities necessary for skin carcinogenesis are independent of environmental stimuli [66,67]. Interestingly, as the development of skin cancer is influenced by skin color, dark-skinned patients with BCC may experience less oxidative and inflammatory impacts, probably due to the antioxidant properties of melanin. Skin cancer is less frequent in individuals with dark skin [68] than Caucasians, and has been associated with impaired systemic oxidative stress values [44]. Lastly, it is worth noting that the interaction between oxidative stress and inflammatory components of the internal exposome also plays a role in other skin conditions, such as psoriasis and atopic dermatitis, increasing the likelihood of flare-ups [66,69].

While our review provides a comprehensive synthesis of systemic alterations in NMSC patients, several limitations should be acknowledged. First, the included studies exhibit methodological heterogeneity, particularly in biomarker assessment techniques, sample types (serum, plasma, and erythrocytes), and analytical protocols, which may contribute to variability in the reported biomarker levels, making direct comparisons between studies challenging. Additionally, the included studies vary substantially in sample size and population characteristics, limiting the generalizability of our findings. Lastly, most studies utilized a cross-sectional design, assessing biomarkers at a single time point rather than tracking their fluctuations over time. This limits our ability to establish causal relationships between systemic oxidative stress and inflammation in NMSC patients.

Our findings highlight new and established biomarkers in NMSC, which with the rise of personalized medicine, might be instrumental in organizing treatment approaches based on individual patient profiles. The systemic values of many previously mentioned biomarkers in NMSC patients allow for easier, more frequent monitoring, which supports assessment and more accessible individualized care. Furthermore, the complex interplay between these biomarkers might explain the scattered results detected in NMSC patients that followed antioxidant supplementation [70]. As systemic medications for the treatment of skin cancer, including NMSC and melanoma, continue to gain ground, these insights may further pave the way for better outcomes in patients with NMSC. To make the most of these advancements, we need a deeper understanding of how systemic biomarkers change over time. Future studies should adopt a longitudinal approach to track these fluctuations and discover their long-term impact. Moreover, standardizing biomarker assessment methods will ensure more reliable comparisons across studies, ultimately strengthening the evidence.

## 5. Conclusions

UV is the primary driver of NMSC occurrence, setting off a cascade of genetic mutations and harmful effects on the skin, including the disruption of redox equilibrium, inflammation, and local immunosuppression. These mechanisms, acting independently or in reinforcing loops, can extend beyond the skin, entering the systemic circulation and having broader health impacts. The NMSC tumor microenvironment further intensifies these processes, releasing biomolecules that contribute to the ongoing cycle of systemic oxidative stress and inflammation. Overall, our findings indicate that in NMSC, systemic health beyond just the skin can be affected, highlighting the importance of a thorough and personalized approach to managing and monitoring patients with the disease.

## Figures and Tables

**Table 1:** Oxidative stress markers.

Category	Oxidative Stress Markers
Free radicals	Cellular ROS/RNS, Peroxides, Hydrogen Peroxide, Superoxide, and Hydroxyl Radicals
Enzymatic antioxidants	Catalase, Glutathione peroxidase (GPx), Glutathione reductase (GR), Superoxide dismutase (SOD), and NAD(P)H: quinone oxidoreductase 1 (NQO1)
Non-enzymatic antioxidants	Glutathione (GSH), Total thiol groups (as total sulfhydryl groups), Co-enzyme Q, NADPH, Uric acid, Bilirubin, and Albumin
Markers of oxidative damage to proteins	CARBS (protein carbonyls), Advanced Glycation End Products (AGE), and Advanced Oxidation Protein Products (AOPP)
Markers of oxidative damage to lipids	Thiobarbituric Acid Reactive Substances (TBARS), Malondialdehyde (MDA), 4-hydroxynonenal (4-HNE), F2-isoprostanes, Lipid Hydroperoxides (LPO), and Oxidized LDL
Markers of oxidative damage to DNA	8-oxo-dGuo and H_2_O_2_-induced DNA damage

**Table 2:** Inflammation/immunosuppression markers.

Category	Inflammation/Immunosuppression Markers
Acute inflammation markers	C-reactive protein (CRP), Erythrocyte Sedimentation Rate (ESR), Procalcitonin (PCT), Serum Amyloid A, alpha-1-acid glycoprotein, Plasma viscosity, Ceruloplasmin, Hepcidin, and Haptoglobin
Cytokines	IL-1β, IL-2, IL-6, TNF-α, Il-17, IL-23, IL-33, IL-10, TGF-β
Soluble immune mediators	Cytotoxic T-lymphocyte associated protein 4 (CTLA-4), Lymphocyte-activation gene 3 (LAG-3), Programmed death-1 (PD-1, PDL-1), and T cell immunoglobulin and mucin-domain containing-3 (TIM-3)
Antimicrobial peptides	Defensin-β and Cathelicidin
Immunosuppression markers	Programmed death-1 (PD-1, PDL-1), IL-10, and TGF-β

**Table 3:** Biochemical parameters.

Category	Biochemical Parameters
Glucose metabolism	Fasting Glucose
Lipid profile	Low Density Lipoprotein (LDL), High Density Lipoprotein (HDL), Total cholesterol, and Total triglycerides (TGs)
Liver function tests	Aspartate Transaminase (AST), Alanine Transaminase (ALT), and Gamma-glutamyltransferase (γ-GT)
Renal function tests	Creatinine and Urea
Hormones	Vit D (expressed in 25-OH-D), Dopamine, Serotonin, Testosterone, and Estrogens
Electrolytes	Calcium, Sodium, Potassium, and Magnesium

**Table 4:** Comparisons of systemic oxidative stress markers in BCC and SCC patients compared with control subjects.

Study Focused on BCC Patients	Specimen	Protocol/Method Used	Oxidative Stress Markers	Results
[32]	Erythrocytes	[31]	Catalase activity (U/mg Hb)	No association
[13]	Plasma	Kit Protocol from Cayman Chemical (Ann Arbor, MI, USA)	Catalase activity (U/mg protein)	Lower
[13]	Plasma	[33]	GPx activity (U/mg protein)	Lower
[13]	Plasma	[34] and Kit Protocol from Cayman Chemical (Ann Arbor, MI, USA)	SOD activity (U/mg protein)	Higher
[13]	Plasma	[35]	NQO1 activity (nmole 2,6-dichloroindophenol reduced/min/mg protein)	Lower
[32]	Erythrocytes	[31]	GSH (μmol/g Hb)	Lower
[13]	Plasma	DTNB enzymatic recycling method following Kit Protocol from Sigma-Aldrich (St. Louis, MO, USA)	GSH (nmol/mg protein)	Higher
[36]	Erythrocytes	[37]	GSH (mg/dL)	Lower
[32]	Plasma	[38]	TAC (mmol DPPH/L)	Lower
[36]	Plasma	[39]	Total thiol groups (mmol/L)	Lower
[32]	Plasma	[40]	TBARS (μmol/L)	Higher
[32]	Plasma	[41]	CARBS (nmol/mg protein)	Higher
[42]	Peripheral blood	[43]	H_2_O_2_-induced DNA damage (mean tail length after H_2_O_2_) (basal mean tail length)	Higher
[44]	Serum	Colorimetric assay, Kit Protocol from Sigma-Aldrich Company, catalog number MAK085	MDA (nmol/mL)	Higher
**Study Focused on SCC Patients**	**Specimen**	**Protocol/Method Used**	**Oxidative Stress Markers**	**Results**
[32]	Erythrocytes	[28]	GSH (μmol/g Hb)	No association
[32]	Erythrocytes	[28]	Catalase activity (U/mg Hb)	Lower
[32]	Plasma	[35]	TAC (mmol DPPH/L)	Lower
[32]	Plasma	[37]	TBARS (μmol/L)	No association
[32]	Plasma	[40]	CARBS (nmol/mg protein)	Higher
[42]	Peripheral blood	[43]	H_2_O_2_-induced DNA damage (mean tail length after H_2_O_2_) (basal mean tail length)	Higher

**Table 5:** Comparisons of systemic inflammation markers in BCC and SCC patients compared with control subjects, including acute inflammatory markers [45], cytokine concentrations, soluble immune mediators, and antimicrobial peptides.

Study Focused on BCC Patients	Specimen	Protocol/Method Used	Inflammation Markers	Marker Significance	Results
**Acute inflammatory markers**					
[46]	Serum	Immunoturbidimetric assay for CRP serum levels (HumaStar 300 analyzer)	C-reactive protein (CRP) (mg/L)	Acute phase reactant protein	Higher
[36]	Serum	Spectrophotometrical assessment with 8 mM 0-dianizidine and absorbance at 500 nm	Ceruloplasmin (U/L)	Acute phase reactant protein–copper phase ferroxidase	Lower
**Cytokines**					
[47]	Serum	ELISA test (Human ELISA kit, Diaclone SAS, France)	IL-2 (pg/mL)	Activates T cell-mediated immune response	Lower
[48]	Serum	ELISA test (Human ELISA kit, Diaclone SAS, France and Human ELISA kit, Cloud-Clone Corp, Katy, TX, USA)	IL-2 (pg/mL)	Mediated immune response	Lower
[48]	Serum	ELISA test (Human ELISA kit, Diaclone SAS, France and Human ELISA kit, Cloud-Clone Corp, Katy, TX, USA)	IL-10 (pg/mL)	Immuno-suppressive cytokine	Higher
[49]	Serum	ELISA test (MyBioSource, San Diego, CA, USA)	IL-17A (pg/mL)	Pro-inflammatory cytokine	Higher
[50]	Serum	ELISA test (Human ELISA kit, Diaclone SAS, France and Human ELISA kit, Cloud-Clone Corp, Katy, TX, USA)	IL-6 (pg/mL)	Pro-inflammatory cytokine	Higher
[51]	Serum	ELISA test (Sigma-Aldrich; St. Louis, MO, USA)	IL-27 (ng/mL)	Not specific inflammatory response	Higher
**Soluble immune mediators**					
[51]	Plasma	Human Immuno-Oncology Checkpoint Protein Panel (Milliplex^®^ MAP Kit (HCKP1–11 K), Merck, KGaA, Darmstadt, Germany)	CTLA-4 (pg/mL)	Μember of the immunoglobulin superfamily that is expressed by activated T cells	Higher
[52]	Plasma	Human Immuno-Oncology Checkpoint Protein Panel (Milliplex^®^ MAP Kit (HCKP1–11 K), Merck, KGaA, Darmstadt, Germany)	LAG-3 (pg/mL)	Ιnhibitory receptor that is highly expressed by exhausted T cells	Higher
[52]	Plasma	Human Immuno-Oncology Checkpoint Protein Panel (Milliplex^®^ MAP Kit (HCKP1–11 K), Merck, KGaA, Darmstadt, Germany)	PD-1, PDL-1 (pg/mL)	Promoter of apoptosis of antigen-specific T cells	Higher
[52]	Plasma	Human Immuno-Oncology Checkpoint Protein Panel (Milliplex^®^ MAP Kit (HCKP1–11 K), Merck, KGaA, Darmstadt, Germany)	TIM-3 (pg/mL)	Inhibitor of Th1 responses and cytokine expression	Higher
**Antimicrobial peptides**					
[53]	Serum	ELISA	Human β-defensin 2 (HBD-2)	Anti-microbial action	Higher
[53]	Serum	ELISA	Cathelicidin	Anti-microbial action	Higher
**Study Focused on SCC Patients**	**Specimen**	**Protocol/Method Used**	**Inflammation Markers**	**Marker Significance**	**Results**
**Acute inflammatory markers**					
[46]	Serum	Immunoturbidimetric assay for CRP serum levels (HumaStar 300 analyzer)	C-reactive protein (CRP) (mg/L)	Acute phase reactant protein	Higher
**Cytokines**					
[54]	Serum	Flow cytometric bead-based immunoassay	Il-6 (pg/mL)	Pro-inflammatory	Higher
[54]	Serum	Flow cytometric bead-based immunoassay	TNF-a (pg/mL)	Pro-inflammatory	Higher
[49]	Serum	ELISA test kit (MyBioSource, San Diego, CA, USA)	IL-17A (pg/mL)	Pro-inflammatory	Higher
[51]	Serum	ELISA test (Sigma-Aldrich; St. Louis, MO, USA)	IL-27 (ng/mL)	Not specific inflammatory response	Higher

## Data Availability

The data described in this study are available upon request from the corresponding author.
